# Computing inbreeding coefficients accounting for unknown parents using pedigree-based metafounders

**DOI:** 10.3168/jdsc.2025-0854

**Published:** 2026-01-30

**Authors:** Che Hsuan Huang, Seijiro Hirama, Toshimi Baba, Junpei Kawakami, Yutaka Masuda, Takeshi Yamazaki, Koichi Hagiya

**Affiliations:** 1 Dairy Cattle Group, Division of Dairy Production Research, Hokkaido Agriculture Research Centre, NARO, Sapporo 062-8555, Japan; 2 Department of Life and Food Science, Obihiro University of Agriculture and Veterinary Medicine, Obihiro 080-8555, Japan; 3 Holstein Cattle Association of Japan, Hokkaido Branch, Sapporo 001-8555, Japan; 4 Rakuno Gakuen University, Ebetsu, Hokkaido 069-8501, Japan

## Abstract

Missing pedigrees result in underestimated numerator relationship and inbreeding coefficients in a closed population. Recursive algorithms have been proposed to derive nonzero inbreeding coefficients accounting for unknown parents but can be computationally ineffective with a deep pedigree. In this report, we proposed a pedigree-based metafounder (MF) approach to calculate nonzero inbreeding. Unknown parents were assigned to MF based on the birth year of their offspring. The MF were assumed to have self-relationships equaling twice the average inbreeding coefficients of the animals with both parents known and born in the same year. The relationships between 2 MF were set as a function (i.e., minimum, harmonic mean, or maximum) of their self-relationships. Inbreeding coefficients were then computed using Sargolzaei and Iwaisaki's (2004) algorithm modified to account for MF, and the relationship matrix of MF was constructed iteratively until convergence. The algorithm was tested and compared with a recursive algorithm using a pedigree of 8.6 million Japanese Holsteins (average longest ancestral path = 11) and a simulated pedigree with 11,550,500 animals across 40 generations. For the Holstein pedigree, one iteration took ∼300 s with the recursive algorithm and 70 s with the proposed algorithm. If the relationships between MF were assumed to equal the maximum of their self-relationships, both algorithms converged to the same results. Simulation results further confirmed that the maximum assumption yielded the best correlation between the estimated and true inbreeding. The discussion highlights how different assumptions regarding the relationships between MF affect the resulting numerator relationship and inbreeding coefficients. With the MF approach, the subsets of the numerator relationship matrix accounting for unknown parents can also be derived quickly.

## Highlights

• Pedigree-based metafounders were used to estimate nonzero inbreeding.
• Different metafounder relationship assumptions result in distinct estimates.
• Estimates align well with Aguilar and Misztal's (2008) results.
• Less computation time per iteration (300 s vs. 70 s) and less memory are required.
• The approach can also be used for mating design (computing future inbreeding).

## Graphical Abstract

**Figure:**
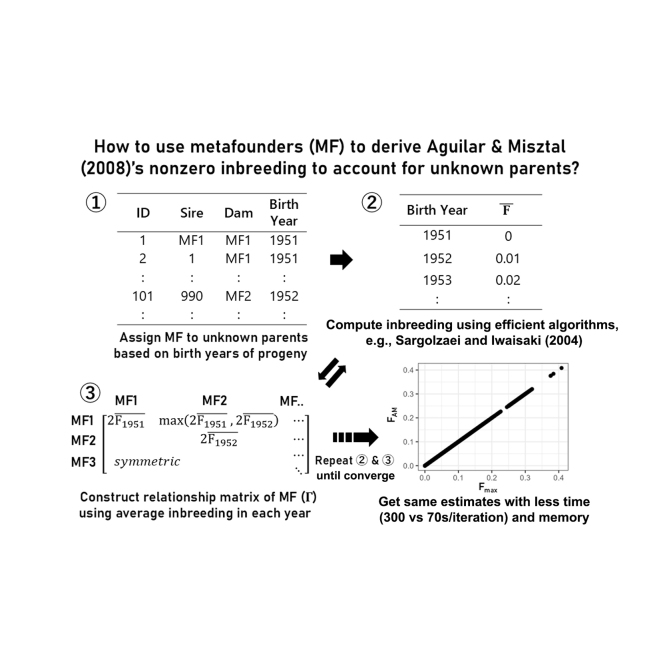
**Summary:** This study introduces a method for estimating inbreeding coefficients when the pedigree is incomplete. Metafounders (MF) are assigned to unknown parents based on the birth year of their offspring, and inbreeding coefficients were computed using a modified version of the Sargolzaei and Iwaisaki (2004) algorithm. The relationships within and between MF are iteratively updated to refine inbreeding coefficients. Compared with the conventional recursive algorithm, which requires 300 seconds (s) per iteration and substantial memory usage, the proposed method completes calculations in 70 s. By testing 3 different assumptions for MF relationships (minimum, harmonic mean, and maximum self-relationships), the study demonstrates how these choices affect inbreeding estimates. The results confirm that this method can produce estimates (Fmax) equivalent to Aguilar and Misztal's (2008) approach (FAM) while allowing for rapid computation of inbreeding and numerator relationship coefficients, resulting in an immediate application to inbreeding management in livestock.

Best linear unbiased predictions for breeding values of livestock rely heavily on a numerator relationship matrix (**A**; Henderson, 1973). The matrix is constructed using pedigree records to describe the additive genetic relationships between animals, or equivalently, the standardized (co)variances of the breeding values. With a complete (co)variance matrix **A**, BLUP efficiently leverages phenotypic data from relatives to estimate breeding values for selection candidates. However, in livestock populations, pedigree records are sometimes missing.

In a closed population of finite size, descendants are expected to be, on average, more inbred and related to other descendants than their ancestors are (Wright, 1931). Consequently, missing pedigrees, especially those of recent animals, lead to underestimation of diagonal and off-diagonal elements (i.e., inbreeding and relationship coefficients) in **A**. This, in turn, introduces bias into the estimated breeding values in BLUP evaluations because unequal amounts of information were accounted for among selection candidates. Additionally, in the single-step genomic BLUP, the genomic relationship matrix (**G**) is integrated with **A** to project the genomic relationships to ungenotyped animals (Legarra et al., 2009). Missing pedigrees compromise the compatibility between **A** and **G**, as the genetic relationships are fully realized by genetic markers but not by incomplete pedigrees [i.e., **A** ≠ E(**G**)], where E(**G**) is the expectation of **G**. If we were to align **G** with **A** using the average of their elements (Vitezica et al., 2011), the genomic-enhanced breeding values would be biased up or down depending on the completeness of the pedigree (Masuda et al., 2022).

To recover the relationships lost due to missing pedigrees, VanRaden (1992) proposed a tabular method to construct a numerator relationship matrix with an unusual assumption. He declared that base animals (i.e., animals with unknown parents) can be inbred and related and implicitly assumed that those born in later generations would be more inbred and related. Practically, the method of VanRaden (1992) has been applied to alleviate the underestimation of inbreeding coefficients due to missing pedigrees. Aguilar and Misztal (2008) later modified this method with a recursive algorithm to improve computational efficiency. Their algorithm had a computational cost of $\sum 2^{p_{l}},$ where *p_l_* is the number of generations until the genetic base for the *l*th animal. This can be inefficient if the pedigree is deep. More recently, Legarra et al. (2015) extended the idea of VanRaden (1992) by introducing metafounders (**MF**). Metafounders are conceptually a generalization of unknown parent groups (Quaas, 1988), accounting for inbreeding, random drift, and relationships among groups of animals (i.e., MF). After defining the relationship matrix of MF (**Γ**), inbreeding coefficients and subsets of **A^Γ^** (an **A** with **Γ** considered) can be computed by more efficient algorithms, such as Meuwissen and Luo (1992) and Colleau (2002), respectively.

Because MF are parents of all base animals in the pedigree, the core of the MF approach is on how to define **Γ**. Garcia-Baccino et al. (2017) have shown that if **G** was constructed with allele frequencies of 0.5, then estimating **Γ** involves estimating the (co)variances of allele frequencies in the base populations. This can be difficult when there are many MF or when MF appear several generations earlier than the genotyped animals (Kudinov et al., 2020; Macedo et al., 2022). An alternative is to estimate the diagonal elements of **Γ** (i.e., the self-relationship of MF) as twice the rate of pedigree-based inbreeding per year plus a constant, **Γ_0_** (Macedo et al., 2022; Legarra and VanRaden, 2023; Legarra et al., 2024). Based on Sorensen and Kennedy's (1983) findings on the structure of **A**, they proposed that the relationship between 2 MF should be the minimum of their self-relationships. However, this conflicts with the work of Aguilar and Misztal (2008), where the relationship between 2 unknown parents was assumed to be twice the maximum of the 2 averages inbreeding of animals with both known parents born in the corresponding years.

In this study, we investigated whether MF can be used to derive the inbreeding coefficients (F_AM_) and the numerator relationship matrix (**Ã**) proposed by Aguilar and Misztal (2008), such that given a specific **Γ**, **A^Γ^** = **Ã**. For calculating inbreeding coefficients, the algorithm by Legarra et al. (2015) involves the Cholesky decomposition (or any square root decomposition) of **Γ**, requiring **Γ** to be at least positive semidefinite (**PSD**), to which we made slight modifications to apply it to any **Γ**. Eventually, inbreeding coefficients were calculated for both the real Holstein and simulated pedigrees to discuss how the assumed relationships between MF (i.e., minimum, harmonic mean, or maximum of their self-relationships) affect the results.

Following previous studies, the unknown parents were assigned to MF based on the birth year (or generation number, if available) of their progeny. The algorithm was iterative. In the first iteration, the inbreeding coefficients were computed with a **Γ** full of zeros. This is equivalent to the original algorithm of Sargolzaei and Iwaisaki (2004), which is similar to but quicker than algorithm of Meuwissen and Luo (1992). In the later iterations, **Γ** was constructed as follows, using the inbreeding coefficients computed in the previous iteration:

$$\begin{array}{c} \Gamma^{k+1}=\\ \left(\begin{array}{cccc} 2\bar{F_{\mathrm{MF}1}^{k}} & f\left(2\bar{F_{\mathrm{MF}1}^{k}}\;,2\bar{F_{\mathrm{MF}2}^{k}}\right) & f\left(2\bar{F_{\mathrm{MF}1}^{k}}\;,2\bar{F_{\mathrm{MF}3}^{k}}\right) & \cdots \\ f\left(2\bar{F_{\mathrm{MF}1}^{k}}\;,2\bar{F_{\mathrm{MF}2}^{k}}\right) & 2\bar{F_{\mathrm{MF}2}^{k}} & f\left(2\bar{F_{\mathrm{MF}2}^{k}}\;,2\bar{F_{\mathrm{MF}3}^{k}}\right) & \cdots \\ f\left(2\bar{F_{\mathrm{MF}1}^{k}}\;,\;2\bar{F_{\mathrm{MF}3}^{k}}\right) & f\left(2\bar{F_{\mathrm{MF}2}^{k}}\;,2\bar{F_{\mathrm{MF}3}^{k}}\right) & 2\bar{F_{\mathrm{MF}3}^{k}} & \cdots \\ \vdots & \vdots & \vdots & \ddots \end{array}\right)\;, \end{array}$$

where **Γ**^k+1^ was the **Γ** in the (k+1)th iteration, whose row *i* column *j* defined the relationship within or between the *i*th and the *j*th MF in the (k+1)th iteration. The self-relationships within MF (i.e., diagonal elements of **Γ**) were set as twice the mean inbreeding coefficients from the previous iteration. Specifically, $\bar{F_{\mathrm{MF}1}^{k}}$ was the mean inbreeding of the kth iteration for animals with known parents and born in the same year (or generation) as the first MF, $\bar{F_{\mathrm{MF}2}^{k}}$ was the corresponding estimate for the second MF, and so on. Because animals in the first generation cannot be inbred, the first MF always has a self-relationship of zero. On the other hand, after one round of iteration, MF in later generations can have nonzero self-relationships, and the animals born from such MF can have nonzero inbreeding. Following VanRaden (1992) and Aguilar and Misztal (2008), the relationships between 2 MF (i.e., off-diagonal elements of **Γ**) were set as a function (*f*) of their self-relationships, which can be the minimum, harmonic mean, or maximum of the self-relationships of the 2 MF.

After defining **Γ**, we considered computing inbreeding coefficients using the algorithm of Sargolzaei and Iwaisaki (2004), with modifications as in Legarra et al. (2015). Unfortunately, their codes involve the Cholesky decomposition of **Γ** and require it to be PSD. To allow a non-PSD **Γ**, we computed the contribution from MF to the inbreeding coefficient of the animal *l* as $L_{l,1:\mathrm{nmf}}\;\Gamma L_{l,1:\mathrm{nmf}}^{'},$ where **L** was a lower triangular matrix containing the proportions of genes that animals derived from their ancestors, nmf was the number of MF, and **L**_l,1:nmf_ was a vector containing the proportions of genes that the animal *l* derived from MF (Legarra et al., 2015). Indeed, the MF part of **L**, **L**_:,1:nmf_, is the **Q** matrix proposed by Quaas (1988), and thus $L_{l,1:\mathrm{nmf}}\;\Gamma L_{l,1:\mathrm{nmf}}^{'}=Q_{l,:}\Gamma Q_{l,:}^{'},$ which gives the contribution from MF to the *l*th diagonal element of **A^Γ^** (Masuda et al., 2022). Eventually, the algorithm iterated to construct **Γ** and compute inbreeding coefficients until convergence.

Two pedigrees were used to test the algorithms. The first was a Japanese Holstein pedigree provided by the Holstein Cattle Association of Japan (Tokyo, Japan), containing Holsteins born from 1951 to 2023 (n = 8,634,903). Missing birth years were estimated assuming a generation interval of 3 years (Aguilar and Misztal, 2008). Pedigrees with parental age conflicts (at least one parent born in the same year as or later than their progeny; n = 48) were removed. The longest ancestral path of the pedigree had a mean of 11 and a maximum of 27. In this pedigree, 0.3% of animals had unknown sires only, 4.8% of animals had unknown dams only, and 12.3% of animals had both parents unknown.

The second pedigree was simulated using QMSim (Sargolzaei and Schenkel, 2009) for 40 generations, with sires averaging 200 progeny each. In the base population (i.e., generation 0), 50,000 dams were randomly mated to 500 sires, each dam producing 2 litters by different sires. From generations 2 to 5, sire and dam numbers increased by 50% of generation 1 per generation, reaching 300,000 animals per generation thereafter. Each generation, the oldest 50% of sires and 30% of dams were replaced by animals with the highest estimated breeding values (heritability = 0.3) from the previous generation. The simulation yielded a pedigree of 11,550,500 animals, with an average inbreeding coefficient of 1.523%. Parentages were then randomly removed at proportions mimicking those observed in the Holstein pedigree.

The algorithm was implemented in Fortran 90, compiled with Intel Fortran Compiler 19.0, and then tested on an Intel Core i3-9100 64-bit processor with a clock speed of 3.60 GHz. The L3 cache size was 6 Mb, and the physical memory size was 64 GB. Three functions (minimum, harmonic mean, and maximum) were compared for estimating MF relationships, resulting in **Γ**_min_, **Γ**_hm_, and **Γ**_max_, and corresponding inbreeding coefficients F_min_, F_hm_, and F_max_. Convergence was achieved when the average absolute difference in inbreeding coefficients between consecutive iterations was less than 10^−6^ (Aguilar and Misztal, 2008). For comparison, we calculated normal inbreeding (F) and F_AM_ using the software INBUPGF90 (Aguilar and Misztal, 2024), which implements the recursive algorithm. Note that the software assumes the relationship between 2 unknown parents equals the maximum of their self-relationships.

For the Holstein pedigree, one iteration took more than 57 h if the coefficients in **Ã** were not memorized (i.e., method 1 in INBUPGF90), and ∼300 s if the elements of **Ã** were properly memorized (i.e., method 2 in INBUPGF90). On the other hand, the computation time of the proposed algorithm was less than 70 s per iteration. For F_min_, F_hm_, F_max_, and F_AM_, convergence was reached at 4, 5, 7, and 7 iterations, respectively. Table 1 shows the correlations and the average absolute differences between the estimates. At the convergence, estimates of F_min_ and F_hm_ were similar, but both considerably differed from (were smaller than) F_AM_. In contrast, F_max_ was numerically the same as F_AM_. The result also confirmed that $A^{\Gamma_{\max}}$ numerically equaled **Ã**.

**Table 1:** Correlations (above diagonal) and average absolute differences (%; below diagonal) between inbreeding coefficients for the Holstein and simulated pedigrees^1^

Item	Holstein pedigree					Simulated pedigree				
	F	F_min_	F_hm_	F_max_	F_AM_	F	F_min_	F_hm_	F_max_	True F
F	—	0.9163	0.9161	0.8636	0.8636	—	0.9595	0.9491	0.7907	0.4948
F_min_	0.3767	—	1.0000	0.9409	0.9409	0.2307	—	0.9992	0.9205	0.6547
F_hm_	0.3856	0.0089	—	0.9424	0.9424	0.2615	0.0309	—	0.9347	0.6751
F_max_	0.7989	0.4222	0.4132	—	1.0000	0.6845	0.4539	0.4230	—	0.8163
F_AM_ or true F	0.7986	0.4219	0.4130	0.0003	—	1.3550	1.1287	1.0979	0.6839	—

1 F = inbreeding coefficients defined by Wright (1922); F_min_, F_hm_, or F_max_ = inbreeding coefficients computed by assuming that the relationship between 2 metafounders is the minimum, the harmonic mean, or the maximum of their self-relationships, respectively; F_AM_ = VanRaden's (1992) inbreeding coefficients computed using INBUPGF90 (Aguilar and Misztal, 2008) for the Holstein pedigree; true F = F computed before removing parentage information for the simulated pedigree.

For the simulated pedigree, INBUPGF90 could not complete the computation due to either substantial computation time (when coefficients were not memorized) or physical memory limitations (when coefficients were memorized). In contrast, the proposed algorithm completed the computation in ∼1,300 s per iteration. Convergence was reached after 7, 9, and 14 iterations for F_min_, F_hm_, and F_max_, respectively. As shown in Table 1, F_max_ showed the strongest correlation with the true F (i.e., computed before removing parentage information), with r = 0.816, compared with F (r = 0.495), F_min_ (r = 0.655), or F_hm_ (r = 0.675). The average absolute difference between the true F and F_max_ was 0.684%, which was considerably smaller than that for F, F_min_, and F_hm_ (1.355%, 1.129%, and 1.098%, respectively).

To investigate how the assumptions on the relationships between MF affected the computed inbreeding coefficients, we plotted F_min_ against F_max_ for animals with at least one parent unknown in the Holstein pedigree (Figure 1). The figure suggests that in animals with both parents unknown, F_min_ and F_max_ correlated well, but in animals with one parent unknown, F_min_ was substantially lower than F_max_. Some additional investigations have shown that the difference between F_min_ and F_max_ increased when the animal was born from parents with substantial differences in pedigree completeness (data not shown).

**Figure 1:**
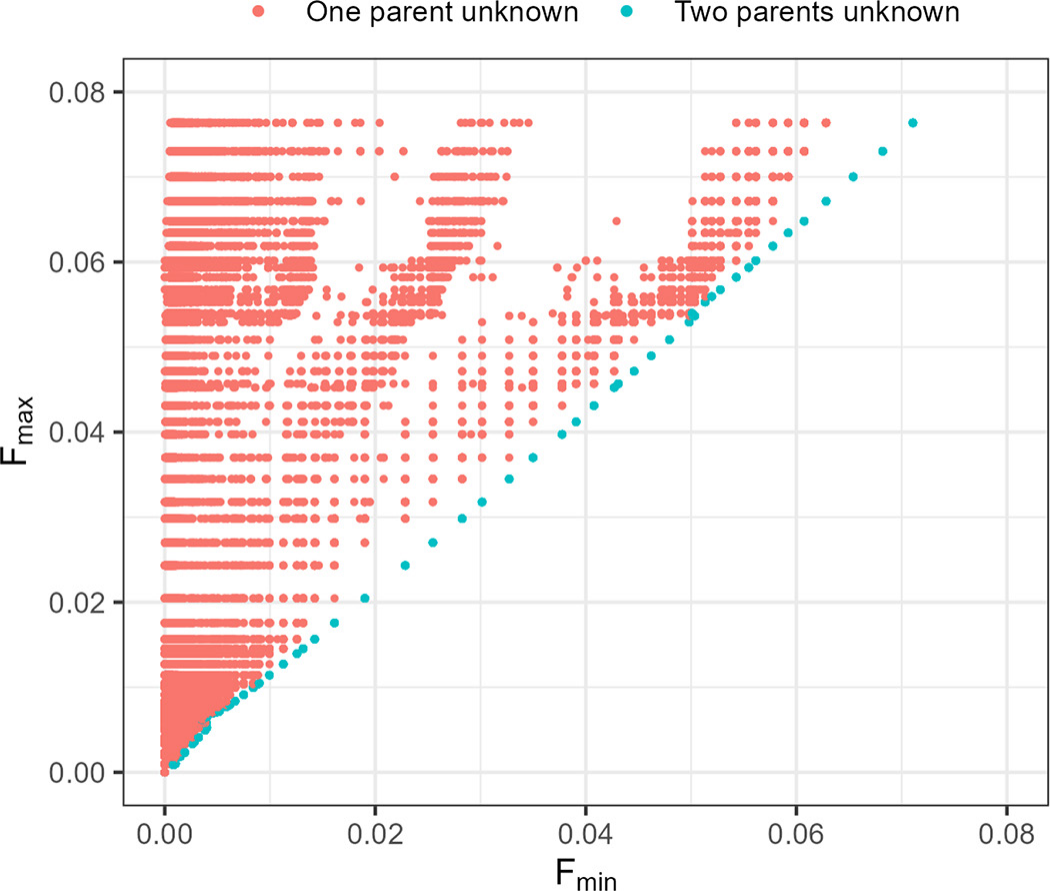
Estimates of F_max_ against F_min_ for animals with at least one unknown parent in the Holstein pedigree.

Table 2 shows an example of the subsets of **A**, $A^{\Gamma_{\min}},$ and $A^{\Gamma_{\max}},$ calculated with the Holstein pedigree using the indirect method (Colleau, 2002). Animals a, b, c, and d were real animals born in 2022 with different pedigree completeness, where a had both parents unknown, b had one parent unknown, and c and d had both parents identified. In normal **A**, animals with both parents unknown (e.g., a) lost all the relationships, and animals with one parent unknown (e.g., b) lost half the relationships with other animals. Using **Γ_min_** did not recover all the relationships. The relationships increased between animals born by the same MF (e.g., between a and b) but not between descendants of different MF (e.g., between a and d). In $A^{\Gamma_{\min}},$ the relationships between past and current MF were underestimated, leading to an underestimation of F_min_, especially in animals whose parents can be traced back to MF of very different generations. In contrast, relationship coefficients in $A^{\Gamma_{\max}}$ were closer to our expectation: for an animal with both parents unknown, its relationships with other animals equaled the average relationship between the animals born in the same year; for animals with more complete pedigrees, their relationships with other animals can be less or more than the average, depending on the pedigree information.

**Table 2:** Subsets of the numerator relationship matrices under different assumptions on the relationships between 2 metafounders (MF)^1^

Item^2^	**A**					$A^{\Gamma_{\min}}$					$A^{\Gamma_{\max}}$				
	MF	a	b	c	d	MF	a	b	c	d	MF	a	b	c	d
MF	0.00	0.00	0.00	0.00	0.00	0.14	0.14	0.07	0.01	0.00	0.15	0.15	0.15	0.15	0.15
Animal															
a	0.00	1.00	0.00	0.00	0.00	0.14	1.07	0.07	0.01	0.00	0.15	1.08	0.15	0.15	0.15
b	0.00	0.00	1.00	0.06	0.06	0.07	0.07	1.00	0.06	0.07	0.15	0.15	1.08	0.14	0.14
c	0.00	0.00	0.06	1.09	0.21	0.01	0.01	0.06	1.10	0.21	0.15	0.15	0.14	1.10	0.22
d	0.00	0.00	0.06	0.21	1.11	0.00	0.00	0.07	0.21	1.11	0.15	0.15	0.14	0.22	1.12

1 Numerator relationship matrices **A** assumed no relationships between or within MFs, $A^{\Gamma_{\min}}$ assumed that the relationship between 2 MF is the minimum of their self-relationships, and $A^{\Gamma_{\max}}$ assumed that the relationship between 2 MF is the maximum of their self-relationships.

2 Animals a, b, c, and d were real animals in the Holstein pedigree born in 2022, and MF corresponds to the MF of 2022. Animal a had both parents unknown, b had one parent unknown, and c and d had both parents known.

In dairy populations, due to finite population size and selective mating, inbreeding coefficients and the relatedness within the existing population (i.e., average coancestry) are expected to increase with time, and animals are usually born by parents existing in the same period. We consider that **Γ**_max_ does a better job than **Γ**_min_ on estimating relationships between animals existing in the same period (Table 2) and thus the inbreeding coefficients of most animals (Table 1). Nonetheless, most research on genetic evaluation with MF was based on a **Γ** with a structure similar to **Γ**_min_ (e.g., Macedo et al., 2022; Legarra and VanRaden, 2023; and Legarra et al., 2024). This may explain why inbreeding coefficients calculated with the approach failed to reflect the true rate of inbreeding (Masuda et al., 2021).

Clearly, constructing **Γ** solely based on pedigree-based F, without estimating the constant **Γ_0_** by maximizing the likelihood function of **Γ** (Legarra et al.,2024), cannot ensure proper alignment between **A^r^** and **G**. In addition, constructing **A^Γ^** using **Γ**_max_ instead of **Γ**_min_ leads to an overestimation of relationships between real animals across generations, particularly for those with unknown parents. Due to this lack of proper alignment between **A^Γ^** and **G**, tuning, the encapsulated unknown parent group or the original MF approaches are still required for single-step genomic BLUP (reviewed by Masuda et al., 2022).

This study presents a straightforward yet efficient algorithm for computing the nonzero inbreeding of unknown parents. Importantly, the results have shown the equivalence of $A^{\Gamma_{\max}}$ and Aguilar and Misztal (2008)'s **Ã**. Consequently, subsets of $A^{\Gamma_{\max}}$ (or equivalently, **Ã**) can be efficiently computed using the indirect method (Colleau, 2002). Overall, the algorithm can help manage inbreeding in populations with incomplete pedigrees. The findings also improve the understanding of the MF approach.
